# Shielding assessment of a mobile electron accelerator for intra‐operative radiotherapy

**DOI:** 10.1120/jacmp.v11i4.3151

**Published:** 2010-09-07

**Authors:** Alexandre S. Krechetov, Donald Goer, Kate Dikeman, Jodi L. Daves, Michael D. Mills

**Affiliations:** ^1^ IntraOp Medical Corp Sunnyvale CA; ^2^ Medical Radiation Physics Inc. Milford OH 45150; ^3^ Department of Radiation Oncology University of Louisville Louisville KY USA

**Keywords:** intra‐operative radiotherapy, electrons, shielding, radiation protection, Mobetron

## Abstract

The purpose of this investigation is to measure, characterize and report stray photon leakage and scatter radiation measurements from the Mobetron, an intra‐operative electron linear accelerator designed for use in an operating room environment. The study is needed due to recent changes to the shielding design of the Mobetron, and also to provide specific information that may be required by regulation in various jurisdictions. An analysis is performed on a number of manufactured units to determine an average 3D stray photon radiation map. This information provides a basis for determining patient‐based load restrictions and/or additional shielding for the operating room. The data presented evaluates the number of procedures for which the Mobetron may be safely operated in a typical unshielded operating room.

PACS number: 87.53.‐j; 87.56.‐v

## I. INTRODUCTION

The purpose of intra‐operative radiotherapy (IORT) is to deliver a single fraction dose of up to 20 Gy to an exposed treatment volume in an operating room while the patient is under anesthesia. The beams may be collimated using applicators with or without bevels. Portions of the field may be blocked using lead, sensitive structures may be relocated or shielded, and bolus may be used to increase the surface dose if needed. The relatively low leakage radiation of the IntraOp Mobetron allows the unit to be used for radiation therapy delivery in an unshielded operating room.^(^ ^1^ ^)^ An assessment of stray photon radiation and patient load has been previously published,^(^ ^2^ ^)^ but recent improvements to the Mobetron shielding design coupled with more detailed measurements on contemporary units make this update desirable.

This study will allow users to establish appropriate workloads for unshielded operating room(s) and select the best operating room(s) to conduct IORT in an unshielded environment.

## II. MATERIALS AND METHODS

### A. Experiment setup for concrete effective TVL measurement

**Figure 1:**
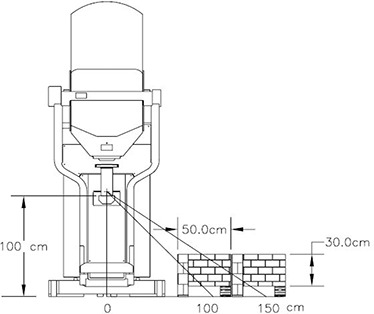
Concrete floor TVL measurement setup.

A set of measurements was taken to evaluate the tenth value layer (TVL) in concrete of the radiation from the Mobetron (IntraOp Medical Corp., Sunnyvale, CA). Concrete blocks (40 cm by 20 cm by 10 cm) of standard density $(2.3g/{\text{cm}}^{3}$) were stacked to simulate various thicknesses of the operating room floor (Fig. 1).

Dosimeters were positioned under the set of concrete blocks. Measurements were taken at 100 and 150 cm from the beam axis for all energies. Blocks were wide enough to ensure that no leakage radiation from the sides would reach the detectors.

### B. Experiment setup for 3D leakage measurement

During leakage data acquisition, the Mobetron was operated at the calibrated dose rate of 10 Gy/min for all nominal electron energies: 4, 6, 9, and 12 MeV. Delivered dose is defined as measured with the 10 cm circular applicator in a water phantom at $D_{\max}$ for the beam energy in use. The Mobetron head was positioned with the beam pointed vertically downwards and moved to the maximum vertical limit of travel. Thus the worst case scenario was implemented with the maximal part of the beam missing the beam stopper.

**Figure 2:**
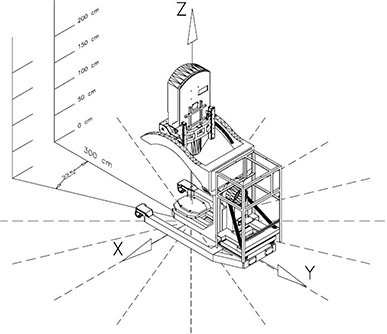
3D exposure distribution measurement setup.

A standard quality assurance fixture was attached to the end of the collimator. The fixture consists of the 10 cm cylindrical aluminum applicator, with 0° bevel, and an attached plastic phantom 13.5 cm in diameter and 8.5 cm long. Five detectors (see detector details below) mounted on a vertical pole at 0, 50, 100, 150 and 200 cm above the floor were used for the measurements (Fig. 2). This setup allowed for a comprehensive 3D map of the leakage, which would be almost impossible to achieve with a standard ion chamber‐based survey meter. Measurements were performed every 22.5° around the Mobetron. The pole was located at 300 cm from z‐axis. Additional measurements were performed at various distances and angles to investigate leakage distribution in space.

### C. Detectors

Mobetron operation results in a combination of photon leakage and scatter, as well as electron scatter. Scattered electrons contribute significantly to the ambient radiation but have a limited range of penetration (on the order of few centimeters in the standard drywall material).

To measure and control the contribution of low penetrating radiation (electrons and low energy X‐rays) to the measured dose, solid state detectors MGP DMC2000XB (MGP Instruments, Smyrna, GA) were chosen. Detectors were calibrated by an independent lab within a year of an experiment. These detectors are capable of separately measuring both $H_{p}$(10) and $H_{p}$(0.07) dose equivalents^(^ ^3^ ^)^ to the accuracy of 1 μSv. The $H_{p}$(10) is the dose equivalent at 10 mm depth in the body at the point of application of the detector. Correspondingly, $H_{p}$(0.07) is the dose equivalent at 0.07 mm depth and usually used as estimated value for the skin dose.

Blocks of gypsum (6 cm thick) were placed in front of each detector for all measurements to attenuate the scattered low penetrating radiation and report only the magnitude of the estimated absorbed dose due to photon radiation (see discussion below).

### D. Data measurement and presentation

A Mobetron‐based coordinate system (CS) has an origin of (0; 0; 0) at the point on the floor under the center of the beam stopper (Fig. 2). Such a convenient point in space as the isocenter cannot be used as the origin of the coordinate system, since the Mobetron doesn't have an isocenter.

Exposure data were measured and analyzed at multiple points around the Mobetron. Experimental data were averaged over four treatment units and then fit using the Fourier series. Estimated relative standard deviation of the presented data is 15%.

The radiation exposure is reported in the three orthogonal planes in CS: X=0,Y=0 and Z=100. These planes are the same as reported in the original Mobetron leakage paper by Daves and Mills,^(^ ^2^ ^)^ and one meter above the floor is a typical height at which the surrounding room leakage surveys are performed.

Measurement results for each of the planes are presented for a 300 cm radius circle, as measured from (0;0;100) every 22.5°. When a 300 cm distance was unachievable (e.g., below the floor), the data were measured as far as possible from the center of the circle and the inverse square law was used to generate the data at 300 cm.

## III. RESULTS

### A. Leakage source and characteristics

To accelerate electrons, the Mobetron uses two X‐band 9.3 GHz collinear accelerators. The first is supplied with constant power and accelerates electrons to 4 MeV. The second is supplied with variable RF power and produces electrons from 4 MeV to 12 MeV at the output. The accelerators are encased in a lead and steel “strong‐back”, significantly reducing the normal guide leakage. In order to deliver the same output dose, the Mobetron gun current is decreased by almost a factor of 10 between 4 MeV and 12 MeV.

It was determined that the source of radiation is distributed along the internal beam path. Two significant regions can be mentioned: the top one between the first and second guide sections, and the bottom one somewhere down the beam line.^(^ ^4^ ^)^ The intensity of the top radiation source is correlated with the gun current and decreases by a factor 10 with an increase in energy, while bottom source increases its intensity according to the well‐known fact that bremsstrahlung in electron beams rises rapidly with energy.

It was found that low penetrating radiation (LPR) contributes significantly to the leakage exposure. Values of the $H_{p}$(10) and $H_{p}$(0.07) measured by uncovered detectors differed by factor of 10 at some measurement points. It was found experimentally by comparing $H_{p}$(10) and $H_{p}$(0.07) that for current Mobetron construction, 6 cm of gypsum board eliminates the effect of LPR completely, while 4 cm of gypsumboard shielding produced numbers 10%–40% higher for $H_{p}$(0.07) relative to $H_{p}$(10).

The transmission of the concrete was measured for the Mobetron radiation using the setup shown in Fig 1. Concrete blocks were piled up on the floor layer by layer. Fig 1 shows last experiment stage when ∼30 cm of concrete was placed over the detectors.

*Effective TVL* values measured in this experiment show how thick a concrete floor should be in order to attenuate the radiation exposure under the floor by a factor of 10. No corrections to obliqueness of the beam path or any other corrections were performed. *Effective TVL* decreases from 41 cm at 100 cm from the beam axis to 28 cm at 150 cm from beam axis.

There was no statistically significant difference detected in attenuation factors for the stray radiation generated by 6, 9 or 12 MeV within error of the measurement. This fact can be explained by the two point geometry of the Mobetron source of radiation. The top source provides most of the radiation at low energies (due to high gun current); the bottom source generates more radiation at higher energies.

The 100 cm data agree reasonably well with NCRP 151^(^ ^5^ ^)^ (as shown in its Table B.2), which reports a concrete TVL for a 10 MeV energy beam as 41 cm and for a 6 MeV beam as 37 cm.

For a 25 cm thick floor, the transmission is 25% at 100 cm from the beam line and 12% at 150 cm from the beam line; for a 15 cm thick floor, it is 43% at 100 cm from the beam line and 22% at 150 cm.

### B. Z‐plane leakage

**Figure 3:**
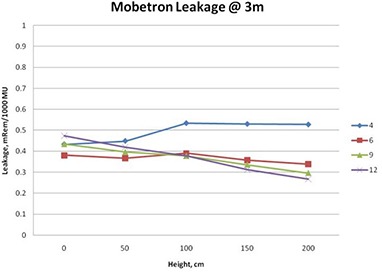
Height dependence of the radiation exposure

For all energies, measured Z‐plane leakage radiation demonstrated significant – sometimes erratic – height dependence for distances less than 3 meters from the z‐axis. At 3 meters, the variation of the leakage with height starts to settle down. Only at distances of 5 meters and greater from z‐axis, does the height dependence of the exposure become not important. Analysis of the data measured at 3 meters from z‐axis for various values of Z (height from the floor) showed that the three highest energies – 6, 9, and 12 MeV –produce similar photon radiation exposure, while 4 MeV consistently generated 20%–40% more exposure for the same delivered dose at all heights but the floor (Fig. 3).

For all values of Z, except directly on or just above the floor, it can be stated with confidence that 4 MeV produces more stray photon radiation than 12 MeV. This can be explained by the two source structure of the Mobetron leakage described above.

**Figure 4:**
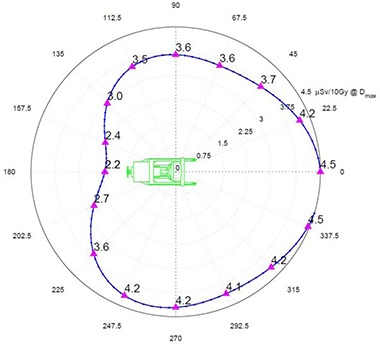
Z=100 plane radiation exposure at 300 cm in μSv per 10 Gy of delivered dose at $D_{\max}$.

Figure 4 shows the Z‐plane exposure at 100 centimeters above the floor (z=100). This projection is useful for determining the potential radiation exposure in rooms and areas adjacent to the OR.

There is about 20% less exposure to the right (positive X) of the unit. This can be attributed to the presence of the magnetron and pulse transformer, which are mounted to the right side of the guide and serve as additional shielding for the leakage radiation.

Otherwise the exposures are quite symmetrical with the expected minimum exposure behind the treatment unit due to its self‐shielding.

In the Z‐plane, exposure averaged over energies has a minimum of approximately 2.2 μSv/10 Gy at the back of the unit and a maximum of approximately 4.5 μSv/10 Gy in the front of the unit.

### C. Floor and ceiling plane exposures

**Figure 5:**
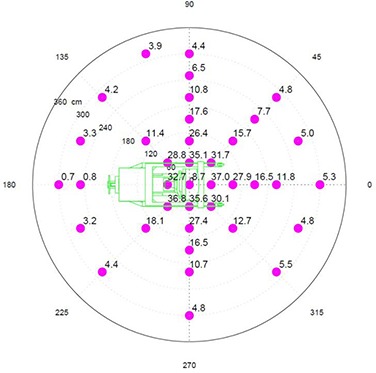
Z=0 floor plane radiation exposure in μSv per 10 Gy of delivered dose at $D_{\max}$.

Radiation exposure of the floor and ceiling differ significantly from the Z‐plane measured at 100 cm above the floor. Exposure diagram showing the distribution of the ambient radiation dose along the floor (z=0) is provided in Fig. 5. The image of the Mobetron on the polar diagram is scaled one‐to‐one in the radial direction so that the back of the Mobetron is approximately at 180 cm from the center and the outer‐most circle is at 360 cm.

Exposure rates just to the side of the beam stopper vary from 32 μSv/10 Gy in the back to 37 μSv/10 Gy in the front, and 8.7 μSv/10 Gy directly under the beam stopper.

The highest level of exposure to the room below should be expected within a 2 meter radius circle centered under the beam stopper and dropping very rapidly as the distance from the center increases. Predicted peak values of the under‐floor leakage can be calculated using concrete effective TVL reported above.

Ceiling radiation exposure (z=330 plane) is maximal right over the beam stopper and slightly in front of the unit. Maximum leakage in this plane is about 3 μSv/10 Gy. Assuming the ceiling above has same thickness of concrete as floor below; exposure above the ceiling will be less than 1 μSv/10 Gy at the maximum and is negligible for all practical purposes.

### D. X‐ and Y‐planes

**Figure 6:**
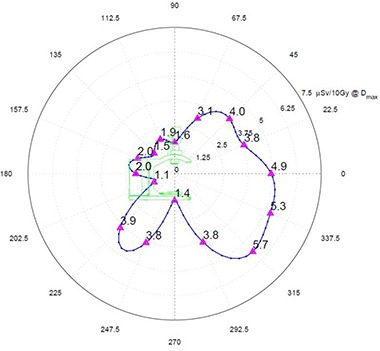
X‐plane radiation exposure at 300 cm in μSv per 10 Gy of delivered dose at $D_{\max}$.

Data for the bottom part of the X‐ and Y‐planes were measured on the floor and extrapolated to 300 cm using the $1/r^{2}$ rule. Peak exposure for the X‐plane (Fig. 6) is 5.7 μSv/10 Gy at 45°. The asymmetry in the X‐plane is due primarily to the self‐shielding of the Mobetron by the cabinet at the back of the unit.

**Figure 7:**
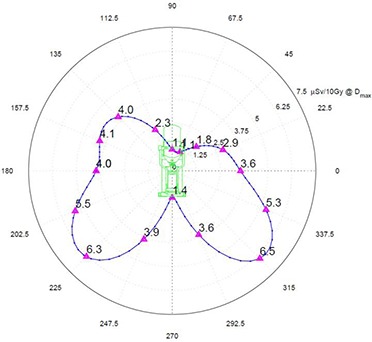
Y‐plane radiation exposure at 300 cm in μSv per 10 Gy of delivered dose at $D_{\max}$.

The Y‐plane (Fig. 7) shows more or less symmetrical exposures with 3.9 μSv/10 Gy and 3.6 μSv/10 Gy just to the side of the beam stopper, and a peak of 6.5 μSv/10 Gy at 45°. Lower values of exposure at the positive X–side are previously discussed in Z‐plane section.

## IV. DISCUSSION

### A. Stray radiation distribution around the operating room

As expected, the floor receives more exposure with increased nominal electron energy, as the more energetic electrons result in more photons generated. In contrast, exposure around the Mobetron is influenced most by the head leakage and increases with gun current in the guide.

Attempts to find a theoretical model which would predict distribution of the radiation at distances closer than 300 cm using either a ‘single point source’, ‘dual point source’ or ‘dual point/linear source’ failed. However, at distances greater than 300 cm, exposure measurements in the Z‐plane can be reasonably described with simple point source model. Measurements at 400, 500 and 700 cm confirmed that beyond 300 cm, leakage radiation from the Mobetron drops as $1/r^{2}$ where distance is calculated from (0;0;100). The height dependence of the exposure is nonsignificant at such distances.

### B. Effect of rotating the unit on the exposure

The effect of rotating the Mobetron from its vertical position must be addressed separately. The Mobetron can rotate a maximum of $\pm 45^{\circ }$ about the y‐axis and a maximum of $\pm 30^{\circ }$ about the x‐axis. As is shown in Fig. 7, the overall peak exposure is directed at 45° downward to the floor. If the Mobetron is rotated 45° about the y‐axis (gantry rotation), then 45° of scatter is projected vertically downwards through the floor, and 45° of scatter is pointing directly toward the side wall.

For the floor below, the 45° scatter is of lower energy than the scatter just missing the beam stopper when the Mobetron is in a vertical position (see discussion about effective TVL above). Thus, even though the exposure on the floor when the Mobetron is rotated to 45° is higher than the exposure when the Mobetron is in the vertical position, the attenuation by the floor is greater for this softer energy scatter.

For adjacent rooms, when the gantry is rotated to its maximum position of 45°, the stray radiation will be highly directional, as can be seen by looking at the Y‐plane exposure for the scatter not absorbed by the beam stopper (Fig. 7). At a distance of 300 cm from the z‐axis, there will be a significant variation of exposure as a function of height. Peak value is about 40% greater than the exposure in front of the unit. Clinically, only a small percentage of the treatments will require such extreme rotations. If as much as 25% of the treatments were at 45°, the exposure to the barrier would only be increased by 10%. As the distance from z‐axis increases, the variation in exposure as a function of height is reduced. Thus, for radiation protection calculations, one can safely use the exposure values with the Mobetron in the vertical position for both adjacent rooms and the floor below.

### C. Allowable workload calculations

These measurements allow the calculation of an allowable workload in cGy/week for all occupied areas surrounding the Mobetron. A worst case scenario for the Mobetron is not clearly defined. As mentioned in the results section, the Z‐plane leakage is maximal for 4 MeV, while floor leakage is maximal for 12 MeV. The decision was made not to include 4 MeV in the calculations since this energy has a very low use factor in practice. Except where mentioned explicitly, all presented data average the exposure rate measurements over 6, 9 and 12 MeV energies, assuming an equal workload contribution and all treatment positions weighted equally.

Table 1 presents workload limits in cGy/week for the adjoining areas on the same floor. Based upon current United States regulatory limits, the allowable exposure level for non‐controlled areas is considered to be 1000 μSv/year, which corresponds to approximately 20 μSv/week. In addition, in a non‐controlled area no more than 20 μSv is allowed during any one hour.

**Table 1:** Workload limits in Gy/week for the non‐controlled adjoining areas on the same floor. Various thickness of lead in the walls can be used.

*Same Floor*		*Distance to Non‐controlled Occupied Area (cm)*		
*Lead (mm)*	*200*	*300*	*400*	*500*	*600*
0	20	40	80	120	170
3	30	60	110	170	240
6	40	90	150	240	340
10	50	100	180	280	410

Table 2 represents workload limits for the occupied areas in the floor below the Mobetron. All calculations in Table 2 were made using an occupancy factor of unity.

**Table 2:** Workload limits in Gy/week for the occupied areas in the floor below the Mobetron as a function of concrete thickness. All calculations were made assuming an occupancy factor of unity.

*Floor Below*		*Distance to Non‐controlled Occupied Area (cm)*			
*Concrete (cm)*	*200*	*300*	*400*	*500*	*600*
15	30	70	130	200	290
25	50	120	220	340	490

These tables can be used as guidelines to quickly determine which operating room(s) and which orientation of the Mobetron may be most suited for operation from the perspective of allowable radiation exposure to surrounding areas.

For the Mobetron, every OR will have a limit on the maximum number of monitor units or beam‐on minutes that can be delivered. Most of the time, Mobetron usage will be limited by the maximum weekly exposure that is allowed. In rare cases where the patient receives substantial treatments to more than one area within short period of time, care should be taken so that the hourly limit is not exceeded.

In some clinical situations, the exposure to the room below can be the limiting factor in workload, especially for hospital buildings with short floor‐to‐floor distances that have thin concrete floors. However, for rooms with floor thicknesses of 25 cm or more, the exposure to the room below is of the same order as the exposures to the adjacent rooms, providing that the floor‐to‐floor height is at least 3.0 meters.

Depending on the floor‐to‐floor distance and floor thickness, the predicted exposure levels in the room below the Mobetron can be comparable and even less than the exposure in the adjacent areas.

### D. Independent stray radiation measurement comparison

The unpublished radiation survey data measured by Anna Petoukhova (Medical Center Haaglanden, The Netherlands) was compared with calculated values using the data in this investigation. Almost all survey numbers are smaller than the predicted values indicating that, in practice, many OR walls will provide some stray X‐ray radiation attenuation, rather than none as assumed in this study.

Several points which are above the predicted value have one common property: there is very thin wall or no wall between the Mobetron and survey meter. Therefore LPR was most likely affecting the measured exposure rate.

## V. CONCLUSIONS

The information in this investigation provides a basis for the determination of monitor unit‐based weekly workload and maximum patient treatment volumes for the Mobetron. Some site‐specific information is required to perform the workload calculations. Current investigation shows that special distribution of the radiation exposure varies insignificantly between the energies with no evident best or worst case. Therefore, any anticipated clinical energy mix will perform similarly relative to the results presented in the paper.

The data and methodology demonstrate that for normal room dimensions, there generally will not be a restriction on patient load. The walls and the floor pose about equal concern for most situations that are encountered. If the areas around OR are occupied and non‐controlled, assuming standard building materials and distances, the facility may be restricted to 150 to 250 Gy per week. Normal patient loads of five to six patients per week, receiving approximately 10–20 Gy, including warm‐up, fit well within the confines of workload limitations calculated here. However, this conservative restriction can be averted if the occupancy of that area is less than one. The measurements and subsequent calculations clearly demonstrate that the Mobetron can be operated in a room with little or no additional shielding.

## ACKNOWLEDGEMENTS

The authors express their appreciation to Richard Simon, Kent Regal, Rick Kelly and James Ferrando for their assistance. This investigation was supported by IntraOp Medical Corp.
